# Unmet financial needs of people with psychosis - a cohort study of people with psychosis, parents, siblings, and controls

**DOI:** 10.1192/j.eurpsy.2025.2222

**Published:** 2025-08-26

**Authors:** J. L. Jansen, J. Hao, R. Bruggeman, G. Investigators, J. Koerts, L. Krabbendam

**Affiliations:** 1 Department of Clinical and Developmental Neuropsychology, University of Groningen; 2 Department of Epidemiology; 3 University Center for Psychiatry, University Medical Center Groningen, Groningen; 4 Department of psychiatry, Amsterdam University Medical Center; 5 Institute for mental health, Arkin; 6 Department of Clinical, Neuro- and Developmental Psychology, Vrije Universiteit Amsterdam, Amsterdam, Netherlands

## Abstract

**Introduction:**

Psychotic disorders have a negative impact on people’s lives, including their financial situation. Limited studies indicate that people with psychosis also have lower subjective evaluations of their financial situation, such as perceived financial needs.

**Objectives:**

This study aimed 1) to examine differences in unmet financial needs between people with psychosis, parents, siblings, and controls, 2) to examine whether family clustering contributes to unmet financial needs, and 3) to examine to what extent substance use, demographic, economic, psychiatric, functional, and cognitive characteristics predict unmet financial needs in people with psychosis.

**Methods:**

Data of the first assessment of people with psychosis (n=956), siblings (n=889), parents (n=858), and controls (n=496) of the Genetic Risk and Outcome of Psychosis study were used. Group differences were assessed with Kruskal-Wallis tests (aim 1). We performed mixed-effect logistic regression analysis and explorative and confirmative ordinal logistic regression analyses for aim 2 and 3, respectively.

**Results:**

People with psychosis reported significantly higher levels of unmet financial need (24%) compared to siblings, parents, and controls (all <10%; table 1). We found no evidence of familial clustering in unmet financial needs. Cannabis and tobacco use significantly and consistently predicted higher levels of unmet financial needs in people with psychosis. Demographic, economic, psychiatric, functional, and cognitive characteristics were no significant predictors.

**Table 1.** Levels of meeting financial needs of people with psychosis, siblings, parents, and controls.

	People with psychosis (n=956)	Siblings (n=889)	Parents (n=858)	Controls (n=496)
Mean (Standard Deviation)	3.2 (1.2)	3.8 (1.0)	4.0 (0.9)	3.9 (.9)
Not at all, % (n)	7.4 (71)	1.5 (13)	1.3 (11)	1.2 (6)
Almost not, % (n)	16.6 (159)	6.9 (61)	2.8 (24)	6.7 (33)
**Unmet financial needs total, % (n)**	24.1 (230)	8.3 (74)	4.1 (35)	7.9 (39)
Average, % (n)	36.9 (353)	29.7 (264)	26.0 (223)	25.8 (128)
Considerable, % (n)	23.6 (226)	35.2 (313)	33.0 (283)	38.3 (190)
Completely, % (n)	15.4 (147)	26.8 (238)	36.9 (317)	28.0 (139)

**Conclusions:**

Relatively high levels of unmet financial needs occur in a heterogeneous group of people with psychosis, especially when they use cannabis or tobacco. Unmet financial needs can have serious consequences for mental health, leisure time, and social activities. Thus, it is pivotal to recognize unmet financial needs, especially combined with substance use, as a stressor for people with psychosis.

**Disclosure of Interest:**

None Declared

